# Immunological cytokine profiling identifies TNF-α as a key molecule dysregulated in autistic children

**DOI:** 10.18632/oncotarget.19326

**Published:** 2017-07-18

**Authors:** Jiang Xie, Li Huang, Xiaohong Li, Hua Li, Yongmei Zhou, Hua Zhu, Tianying Pan, Keith M. Kendrick, Wenming Xu

**Affiliations:** ^1^ The Third People’s Hospital of Chengdu, Affiliated Hospital of Southwest Jiao Tong University Medical School, Chengdu, China; ^2^ Department of Clinical Medicine, Southwest Medical University, Luzhou, China; ^3^ National Office for Maternal and Child Health Surveillance of China, Department of Obstetrics, West China Second University Hospital, Key Laboratory of Birth Defects and Related Diseases of Women and Children, Ministry of Education, Sichuan University, Chengdu, China; ^4^ Department of Obstetrics and Gynecology, West China Second University Hospital, Key Laboratory of Birth Defects and Related Diseases of Women and Children, Ministry of Education, Sichuan University, Chengdu, China; ^5^ Key Laboratory for Neuroinformation, Center for Information in Medicine, University of Electronic Science and Technology of China, Chengdu, China; ^6^ Joint Laboratory of Reproductive Medicine, SCU-CUHK, West China Second University Hospital, Sichuan University, Chengdu, China

**Keywords:** TNF-α, cytokines, autism, THRIL mRNA, LincRNA

## Abstract

Recent studies have suggested that the etiology of autism spectrum disorder (ASD) may be caused by immunological factors, particularly abnormalities in the innate immune system. However, it is still unclear which specific cytokines may be of most importance. The current study therefore investigated which cytokines showed altered concentrations in blood in ASD compared with healthy control children and which were also correlated with symptom severity. Our study sample included 32 children diagnosed with ASD and 28 age and sex-matched typically developing children. Autism symptoms were measured using the Autistic Behavior Checklist (ABC) and blood samples were taken from all subjects. We used Milliplex cytokine kits to determine serum concentrations of 11 Th1, Th2 and Th17 related cytokines. Additionally, expression of THRIL (TNFα and hnRNPL related immunoregulatory LincRNA), a long non-coding RNA involved in the regulation of tumor necrosis factor- α (TNF-α), was determined using real–time PCR. Of the 11 cytokines measured only concentrations of TNF-α (p=0.002), IL-1β (p=0.02) and IL-17a (p=0.049) were significantly increased in ASD children compared to typically developing controls, but only TNF-α concentrations were positively correlated with severity of ASD symptoms on all 5 different ABC sub-scales and were predictive of an ASD phenotype (area under the curve = 0.74). Furthermore, THRIL RNA expression was significantly decreased in ASD children. Our results provide further support for altered innate immunity being an important autism pathogenic factor, with autistic children showing increased blood TNF-α concentrations associated with symptom severity, and decreased expression of the THRIL gene involved in regulating TNF-α.

## INTRODUCTION

Autism spectrum disorder (ASD) is a neurodevelopmental disorder with reports of increasing prevalence during the last decades [1–3] and estimated to affect around 1% of individuals, equating to over 50 million worldwide [4]. While autistic symptoms are variable the three core characteristics presented are problems with social interactions, language communication and repetitive behavior, with other common features being depression, anxiety and lack of attention [5–7]. It has now been widely accepted that the major pathogenesis of ASD includes contributions from both genetic and environmental factors, with epidemiological results from twin studies showing that at least 60% percent of etiology can be linked to gene alterations [8], although it seems likely that multiple genes are involved. There has therefore been an increasing emphasis on identifying potential biochemical markers which may assist in early diagnosis of the disorder in order to allow early therapeutic intervention.

Recent studies have provided increasing evidence that immunological factors, particularly pro-inflammatory ones, may play an important role in the etiology of autism [9–12] and indicate that abnormalities in the innate immune system could be a predominant feature of ASD. Cytokines as a broad category of small proteins secreted from immune cells like macrophages and T lymphocytes are critical mediators of the immune response. T cells include several subtypes, such as Th1, Th2 and Th17 and a number of studies have reported altered concentrations of a range of different cytokines in blood samples from ASD patients [13–15]. In terms of their potential use as an early biomarker for ASD recent studies have also reported cytokine changes can be detected in both amniotic fluid [16, 17] and neonatal blood [18] samples. Currently, increased concentrations of interleukins (IL - IL-1β, IL-4, IL-6) and tumor necrosis factor-α (TNFα) have also commonly been reported in ASD [13–15, 19–23], although it is still unclear which cytokines are the most reliable biomarkers and which are also associated with symptom severity. It is therefore important to establish in more detail which of the many different candidate cytokines related to innate immunity and Th1, Th2 or Th17 show the most consistent alterations in blood concentrations in ASD children and are also associated with symptom severity. In addition, it is important to establish whether altered concentrations of specific candidate cytokines related to ASD are associated with corresponding changes in the expression of genes involved in their regulation.

In the current study, we have measured blood concentrations of 11 different Th1,2 and 17 cytokines in a sample of ASD children and typically developing children from the southwest of China. The study particularly identified TNF-α as showing altered concentrations in ASD children which were associated with symptom severity and so in an additional preliminary analysis we also measured altered expression of the recently cloned long noncoding RNA, THRIL (TNFα and hnRNPL-related immunoregulatory large intergenic non-coding RNA (lincRNA)), which is involved in the regulation of TNF-α via heterogeneous nuclear ribonucleoprotein L (hnRNPL) [24].

## RESULTS

### Demographic comparisons

While the mean age of the participants did not differ significantly between the autism group and the TD group (t-test, t=1.979, p=0.053), the TD group children tended to be slightly older the ASD children (see Table 1). However, none of the blood cytokine concentrations showed any relationship with age. There were no significant differences in gender composition of the two groups (p=0.192).

**Table 1:** Sociodemographic characteristics in the study groups

	ASD N=32	TD N=28	Value	P
Mean Age, years				
Children	5.51±1.280	6.11±1.040	1.979	>0.05
Birth Weight (g)	3140.63±548.670	3012.50±646.733	0.830	0.410
Sex, N (%)				
Male	29(90.63%)	22(78.57%)	1.702	0.192
Female	3(9.38%)	6(21.43%)		

### Autism symptom (ABC) scores

All the children in the ASD and TD groups were assessed using the ABC. Total scores for the two groups together with those in each of the ABC’s five dimensions are shown in the Table 2, with the ASD group scoring significantly higher than the TD group across all five dimensions and the total score (p<0.001 in all cases).

**Table 2:** Autism Behavior Checklist (ABC) total and subscale scores in ASD and TD groups

	ASD N=32		TD N=28		Value	P
	Mean	SD	Mean	SD		
Sensory behavior	12.41	4.211	0.46	1.427	14.292	<0.001
Social Relating	19.94	6.657	1.43	3.271	13.359	<0.001
Body and object use	16.16	7.692	5.11	6.713	5.887	<0.001
Language	15.75	6.011	1.71	3.004	11.186	<0.001
Social and adaptive	14.25	4.711	3.21	4.14	9.574	<0.001
Total score	78.5	15.417	11.89	14.39	17.22	<0.001

### Cytokine concentrations in the serum of ASD and TD children

**Figure 1:**
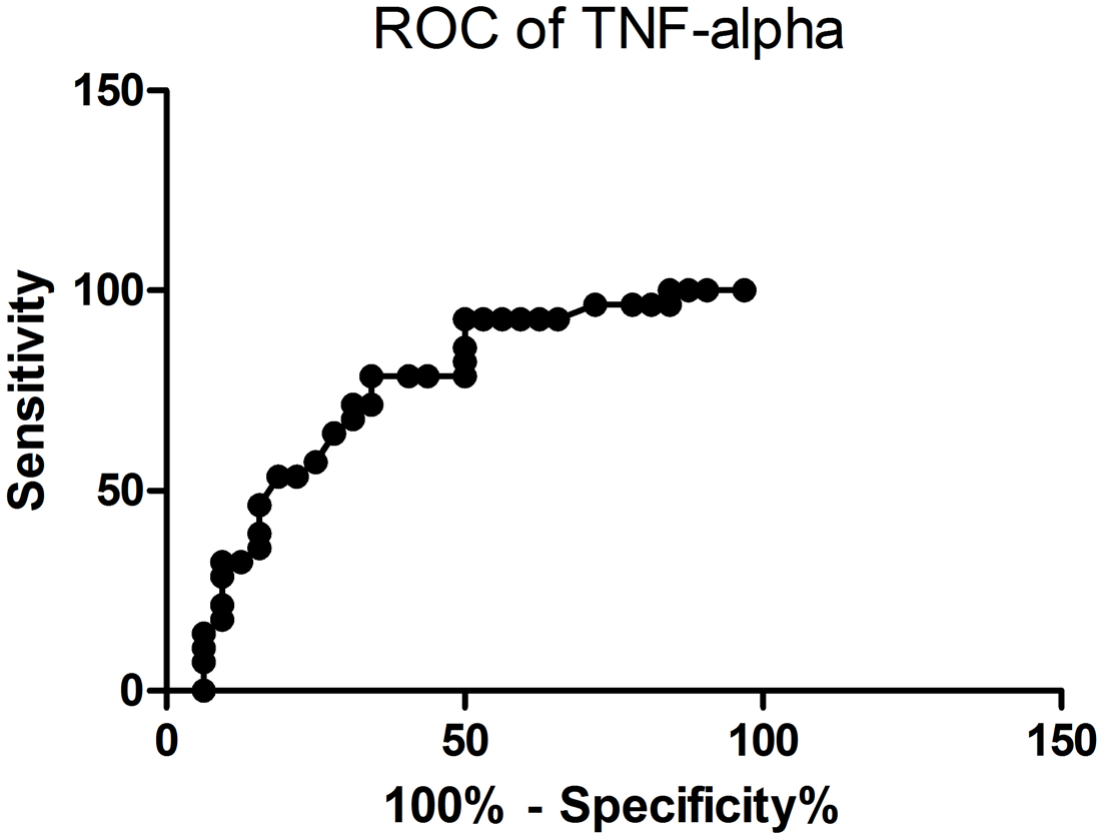

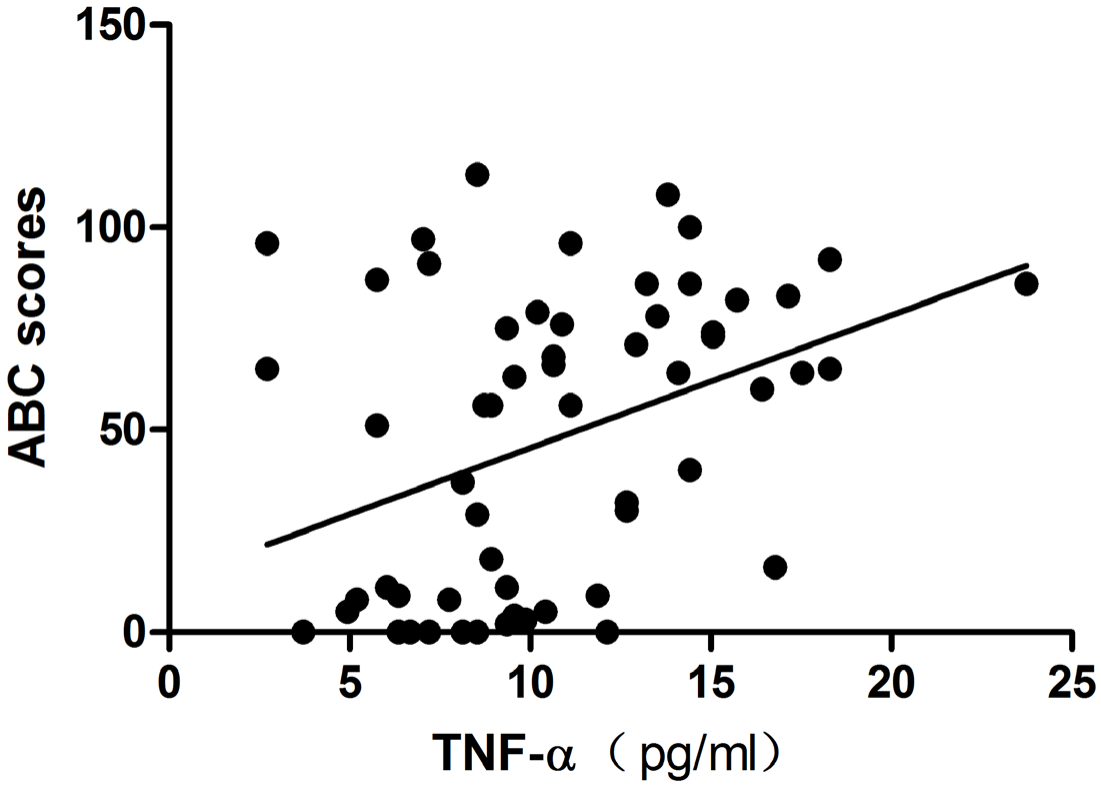
**(A)** Area under the curve (AUC) plot for prediction sensitivity of blood TNF-α concentrations (pg/ml) and ASD vs TD classification (AUC = 0.7411; p<0.01). **(B)** Regression plot showing significant a positive correlation between TNF-α concentrations (pg/ml) and total Autism Behavior Checklist (ABC) scores (r=0.39; p=0.002).

Concentrations of each of the cytokines measured were compared statistically between the ASD and TD groups using t-tests. Table 3 shows serum concentrations of the 11 cytokines we screened in ASD and TD children. TNF-α (p=0.002), IL-1β (p=0.023) and IL-17α (p=0.048) were significantly increased in the ASD group, although only changes in TNF-α survived correction for multiple testing (Bonferroni adjusted p=<0.0045 i.e. 0.05/11). When we performed a receiver operating characteristics (ROC) analysis on the study sample for TNF-α, this revealed a good discrimination sensitivity between ASD and TD subjects (area under the curve = 0.7411±0.065, p<0.01 – see Figure 1A).

**Table 3:** Blood cytokine concentrations in the ASD and TD groups

	ASD N=32		TD N=28		Value	P
	Mean	SD	Mean	SD		
IL-17F ng/ml	0.11	0.602	0.02	0.033	0.848	0.400
IFN-γ pg/ml	7.81	6.010	6.85	3.484	0.742	0.461
IL-10 pg/ml	0.68	0.965	0.51	0.969	0.686	0.496
IL-17A pg/ml	6.92	2.982	5.65	1.761	1.963	0.048*
IL-22 ng/ml	1.30	7.245	0.24	0.625	0.771	0.444
IL-1B pg/ml	3.09	1.177	2.53	0.608	2.252	0.023*
IL-2 pg/ml	3.98	1.832	3.30	1.132	1.696	0.095
IL-21 pg/ml	13.34	8.717	9.76	6.390	1.792	0.078
IL-4 ng/ml	0.04	0.026	0.03	0.023	0.559	0.578
IL-6 pg/ml	3.78	5.099	1.90	2.694	1.749	0.086
TNF-α pg/ml	12.15	4.627	8.77	3.168	3.259	0.002*

* P < 0.05 between the ASD and control groups.

### Relationship between cytokine concentrations and ABC scores

Spearman correlation analysis showed that of the 3 cytokines exhibiting significant differences between the ASD and TD groups only TNF-α concentrations were positively correlated with all five subscales of the ABC (rs=0.29 – 0.41, all ps<0.025) as well as the total scores (r=0.394, p=0.002) in the whole study sample (see Table 4). The regression plot in Figure 1B shows a linear correlation across the total ABC scores in the TD and ASD subjects.

**Table 4:** Correlation analysis of cytokine levels and Autism Behavior Checklist (ABC) scores for children in both groups

	n	Sensory behavior		Social relating		Body and object use		Language and communication		Social and adaptive skills		Total score	
		r	p^c^	r	p^c^	r	p^c^	r	p^c^	r	p^c^	r	p^c^
TNF-α	60	0.290	0.025*	0.315	0.014*	0.412	0.001**	0.388	0.002**	0.400	0.002**	0.394	0.002**
IL-1β	60	0.235	0.071	0.159	0.224	0.251	0.054	0.258	0.046*	0.132	0.313	0.223	0.087
IL-17A	60	0.175	0.180	0.133	0.313	0.240	0.065	0.224	0.085	0.142	0.281	0.184	0.158

*P < 0.05) between the ASD and control groups.

** P < 0.01) between the ASD and control groups.

To further determine whether TNF-α could be used as an independent predictor of autistic behavior symptoms, a stepwise multiple linear regression model was used. The result shows that only TNF-α was an independent predictor of the total score and all 5 subscales of the ABC (see Tables 4-5).

**Table 5:** Relationship between cytokines and Autism Behavior Checklist (ABC) scores

	Unstandardized coefficients		Standardized coefficients	T	P	Model adjusted R^2^
	B	SE	Β			
Model 1. Sensory behavior score as dependent variable						
1. Constant	-2.144	3.114		-0.689	0.494	0.106
2. Gender	4.690	2.339	0.248	2.005	0.050	
3. TNF-α	0.472	0.195	0.300	2.427	0.018	
Model 2.Social relating score as dependent variable						
1. Constant	-2.966	4.898		-0.606	0.547	0.108
2. Gender	7.886	3.679	0.265	2.144	0.036	
3. TNF-α	0.715	0.306	0.289	2.338	0.023	
Model 3. Body and object use score as dependent variable						
1. Constant	0.350	4.128		0.085	0.933	0.119
2. Gender	2.542	3.101	0.101	0.820	0.416	
3. TNF-α	0.803	0.258	0.383	3.114	0.003	
Model 4. Language and communication score as dependent variable						
1. Constant	-2.971	3.804		-0.781	0.438	0.153
2. Gender	4.463	2.858	0.188	1.562	0.124	
3. TNF-α	0.792	0.238	0.402	3.334	0.002	
Model 5. Social and adaptive skills score as dependent variable						
1. Constant	-1.668	3.118		-0.535	0.595	0.175
2. Gender	4.09	2.342	0.208	1.746	0.086	
3. TNF-α	0.69	0.195	0.421	3.54	0.001	
Model 6. Total ABC score as dependent variable						
1.Constant	-9.445	16.096		-0.587	0.56	0.176
2.Gender	23.657	12.09	0.232	1.957	0.055	
3.TNF-α	3.476	1.005	0.411	3.457	0.001	

### The expression of long noncoding THRIL mRNA in ASD and TD children

**Figure 2:**
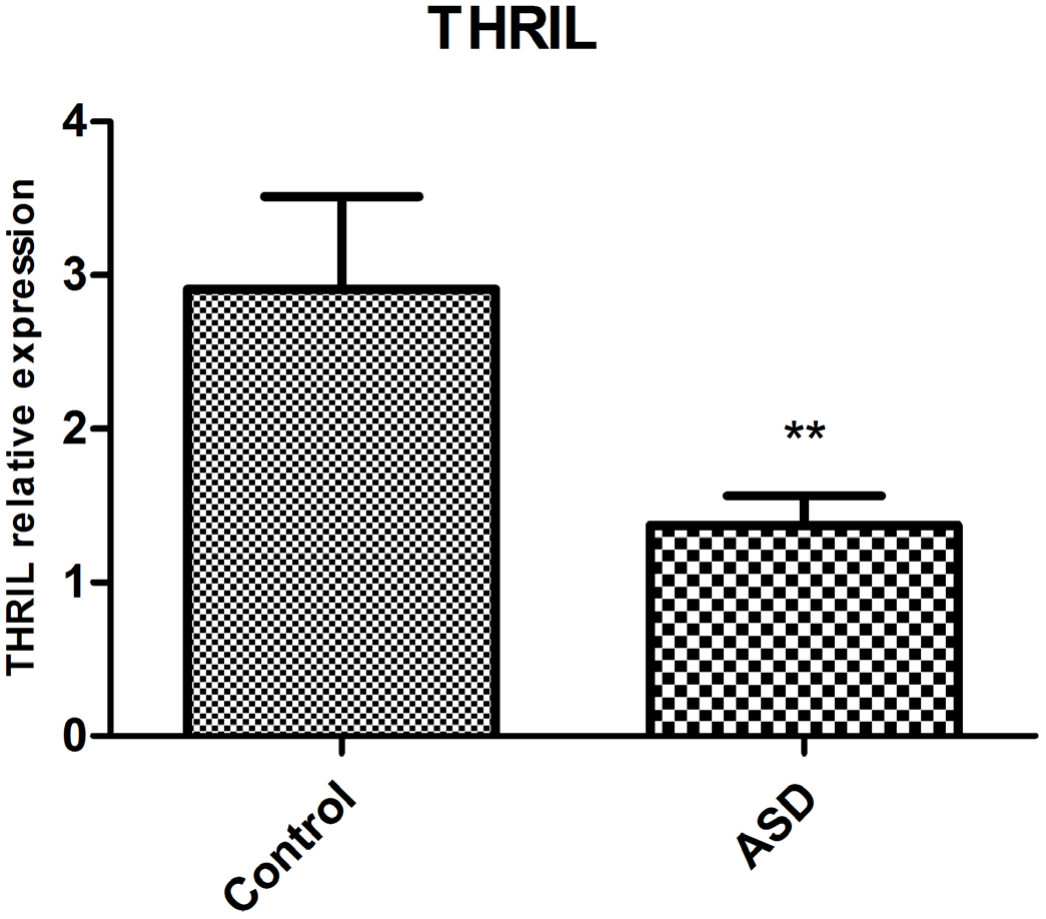
Mean±SEM THRIL mRNA expression (arbitrary units) in blood samples taken from ASD (n=10) and TD (n=8) subjects (t-test **p<0.01).

THRIL is a recently identified long noncoding RNA which could negatively regulate TNF-α expression in THP1 macrophages. Therefore the THRIL-TNF-α regulatory pathway could be altered in ASD patients. As a result of our TNF-α findings we therefore additionally decided to investigate a possible association with THRIL expression. The results showed that THRIL expression was also significantly (t-test, p<0.01) decreased in the ASD compared to TD children (Figure 2, n= 10 for ASD group and n=8 for TD subjects).

## DISCUSSION

Our study has provided further support for the potential use of immunological measures as ASD biomarkers with blood concentrations of TNF-α and expression of its associated long noncoding RNA, THRIL, offering the most promising candidates, although IL-1β may also be of importance. Critically, TNF-α concentrations were positively correlated with severity of autistic symptoms across both TD and ASD subjects, and preliminary evidence for decreased THRIL mRNA expression suggests that this lincRNA could also be associated with ASD pathogenesis.

Although a recent meta-analysis has reported that mainly IL-1β and IL-6 levels are significantly increased in ASD [14], a number of studies have reported that TNF-α concentrations are also increased in blood, cerebrospinal fluid or post-mortem brain tissue [13–15, 19–23]. Our study has confirmed these latter findings in our cohort of subjects and importantly that blood concentrations of TNF-α are positively associated with ASD symptom severity, thus indicating that TNF-α could be an important cytokine biomarker in ASD. It is still unclear whether the increased levels of TNF-α and other cytokines are a reflection of a pro-inflammatory status in ASD children or dysfunctional immune regulation occurring in autism pathogenesis. However, recent animal model and epidemiological studies have indicated that infection, especially during pregnancy, could be critically involved in the etiology of autism [27–31]. Maternal [17] and neonatal [18] cytokine profiles are also associated with the subsequent development of ASD, suggesting that maternal and/or neonatal infections could contribute as well. Animal studies have additionally shown that immunological challenges with lipopolysaccharide (LPS) or PolyI/C treatment during pregnancy can lead to autism-like symptoms [32–33].

How could a maternal or neonatal infection/immune response lead to brain dysfunction and subsequent ASD symptoms? TNF-α is mainly secreted by macrophages and is broadly involved in the innate immune response through the TNF receptor (TNFR) and activates NF-κb to regulate down-stream genes. TNF- α, as well as IL-1’s and IL-6, can all cross from the peripheral blood into the brain to directly affect brain function via their receptors [34]. In humans, preeclampsia and placental ischemia, which are associated with increased incidence of ASD [35], also increase TNF-α concentrations and this in turn can alter the permeability of the blood-brain barrier and influence brain development and function [35]. Similarly, TNF-α can influence the intestinal epithelial barrier and it is possible that elevated concentrations may contribute to gastrointestinal problems that are often associated with ASD [36], although a recent study did not find such an association [13]. Additionally, within the brain itself, microglial cells can secrete TNF-α [37, 38] and microglial dysfunction may lead to ASD pathogenesis, although further studies are clearly required to establish whether microglial secreted TNF-α contributes to ASD. Finally, inflammatory cytokines such as TNF-α are important modulators of neurogenesis [39], and altered neocortical neurogenesis has been implicated in ASD [40].

We have provided the first preliminary evidence that expression of the long noncoding RNA, THRIL, is decreased in ASD, although our results need to be confirmed in a larger cohort of subjects. A recent study has shown that increased THRIL expression in macrophages following LPS stimulation may regulate TNF-α expression through an epigenetic mechanism [24]. However, TNF-α can reduce THRIL expression via a negative feedback action [24], and since we found that its expression is decreased in ASD children this might suggest that it is the increased TNF-α which is responsible for. However, while this requires further research, overall our findings generally support an involvement of TNF-α signaling in ASD, although whether via macrophages or microglial cells, or both, remains to be established.

The TNF-α mediated signaling pathway has been extensively studied and several inhibitors or monoclonal-antibodies have been successfully used for the treatment of rheumatoid arthritis and inflammatory bowel disease [41, 42]. Although TNF-α has been implicated in several brain disorders, such as Alzheimer’s disease [43], it has yet to be explored as a specific neurotherapeutic target in ASD [23, 44, 45]. However, several anti-inflammatory drugs have been shown to have some therapeutic benefit in ASD children [45].

Our study also found evidence for a significant (uncorrected) increase of IL-17A level in ASD children. Th17 related cytokines have also been found to be increased in ASD children by other studies, and a recent study using a mouse model has reported that maternal infection caused Th17 over-activation and autistic-like symptoms, which could be blocked by IL-17 antibody [46]. The clinical significance of Th17-related cytokines in ASD therefore also merits further investigation.

In summary, our study further supports a role for altered TNF-α and its regulation in ASD as the most sensitive cytokine biomarker and for the first time implicate the non-coding LincRNA THRIL which contributes to its regulation. Additionally, TNF-α and its regulation may hold potential as a future therapeutic target.

## MATERIALS AND METHODS

### Subjects

This study was reviewed and approved by the Human Ethics Committee of the third people’s Hospital. The subjects in the autism group (n=32) were recruited from the three training schools for autistic children in Chengdu. After obtaining written informed consent from the children’s parents or legal guardians, the children in the autism group were diagnosed in accordance with DSM-IV-TR criteria for ASD. The autism Behavior Checklist (ABC) was also used to confirm the diagnosis of ASD and provide a measure of symptom severity. The subjects in the typically developing (TD) control group (n=28) were recruited from volunteers at a primary school in the same city. The demographic data for the two groups are shown in Table 1.

### Autism Behavior Checklist (ABC)

The autism behavior checklist (ABC) questionnaire was used as an instrument to quantify symptom severity in the ASD group. The ABC consists of 57 items and 5 scales (1) Sensory behavior, (2) Social relating, (3) Body and Object use, (4) Language and communication (5) Social and adaptive skills [25]. Each item is rated on a 4-point scale ranging from 0 to 3 (reflecting the degree of severity) as assessed by a researcher interviewing the child’s parents/caregiver. A widely used Chinese translation of the ABC was used [26] and in the current cohort of subjects showed good internal consistency (Cronbach’s α = 0.892).

### Blood sampling and measurement of cytokine concentrations

A fasting blood sample (5 mL) was drawn from each ASD and TD subject and the blood was centrifuged at 1200g for 10 min, within 2 hours. The serum was then aliquoted into 500-μL straws and frozen at -80°C until use. Serum concentrations of Th1/Th2 and Th17 related cytokines, were measured by MILLIPLEX MAP custom Human Magnetic Bead Panel Kits (Millipore, Billerica, MA, USA) based on the Luminex xMAP technology. Concentrations of the cytokines (pg/ml or ng/ml) were calculated using a standard curve. Two replicate quality control samples were run with each assay (replicate QC1 samples, low level; replicate QC2 samples, high level). The coefficients of variation (CVs) of replicate quality control samples were <10% for all cytokines.

### Analysis of THRIL mRNA

Total RNA was extracted from whole blood RNAiso Plus (D9108A; TaKaRa, Dalian, China), and the RNA concentration was measured by ultraviolet spectroscopy (Nanodrop 2000; Thermo, Massachusetts, USA). Total RNA (500 ng) was used for reverse transcription with a PrimeScriptRT reagent kit and gDNA Eraser (DRR047A; TaKaRa). Primers were designed and synthesized by Ribobio (Guangzhou, China); β-actin was used as an endogenous control for gene expression analysis. The sequences of the PCR primer pairs for each gene are as follows: Forward: 5’-AACTCCTGACCTCAGGTGATCCAT-3’; Reverse:5’-AAGGGAGTTTCAGAAGGTGTGGCT-3’. For quantitative real-time PCR (qRT-PCR), we used a real-time PCR instrument (Thermal Cycler Real Time System, ABI7500) with the SYBRPremix Ex Taq II (DRR820A; TaKaRa). PCR cycling conditions included pre-denaturing at 95°C for 10 min, followed by 40 cycles (94°C for 15 s, 60 °C for 1 min, 72°C for 15 s) and by an extension at 72°C for 30 s. The mean threshold cycle (Ct) values were normalized to β-actin, and the relative mRNA levels of THRIL were analyzed by the ΔΔC_T_ method. Samples were analyzed in triplicate.

## Abbreviations

ASD: autism spectrum disorder
ABC: Autism Behavior Checklist
